# Proteomic and metabolic profile analysis of low-temperature storage responses in *Ipomoea batata* Lam. tuberous roots

**DOI:** 10.1186/s12870-020-02642-7

**Published:** 2020-09-21

**Authors:** Peng Cui, Yongxin Li, Chenke Cui, Yanrong Huo, Guoquan Lu, Huqing Yang

**Affiliations:** School of Agriculture and Food Science, Zhejiang Agriculture & Forestry University, Hangzhou, 311300 China

**Keywords:** Sweetpotato, Low-temperature storage, Proteometabolomic, Starch metabolism, Chilling tolerance

## Abstract

**Background:**

Sweetpotato (*Ipomoea batatas* L.) is one of the seven major food crops grown worldwide. Cold stress often can cause protein expression pattern and substance contents variations for tuberous roots of sweetpotato during low-temperature storage. Recently, we developed proteometabolic profiles of the fresh sweetpotatoes (cv. Xinxiang) in an attempt to discern the cold stress-responsive mechanism of tuberous root crops during post-harvest storage.

**Results:**

For roots stored under 4 °C condition, the CI index, REC and MDA content in roots were significantly higher than them at control temperature (13 °C). The activities of SOD, CAT, APX, O_2_^.-^ producing rate, proline and especially soluble sugar contents were also significantly increased. Most of the differentially expressed proteins (DEPs) were implicated in pathways related to metabolic pathway, especially phenylpropanoids and followed by starch and sucrose metabolism. L-ascorbate peroxidase 3 and catalase were down-regulated during low temperature storage. α-amylase, sucrose synthase and fructokinase were significantly up-regulated in starch and sucrose metabolism, while β-glucosidase, glucose-1-phosphate adenylyl-transferase and starch synthase were opposite. Furthermore, metabolome profiling revealed that glucosinolate biosynthesis, tropane, piperidine and pyridine alkaloid biosynthesis as well as protein digestion and absorption played a leading role in metabolic pathways of roots. Leucine, tryptophan, tyrosine, isoleucine and valine were all significantly up-regulated in glucosinolate biosynthesis.

**Conclusions:**

Our proteomic and metabolic profile analysis of sweetpotatoes stored at low temperature reveal that the antioxidant enzymes activities, proline and especially soluble sugar content were significantly increased. Most of the DEPs were implicated in phenylpropanoids and followed by starch and sucrose metabolism. The discrepancy between proteomic (L-ascorbate peroxidase 3 and catalase) and biochemical (CAT/APX activity) data may be explained by higher H_2_O_2_ levels and increased ascorbate redox states, which enhanced the CAT/APX activity indirectly. Glucosinolate biosynthesis played a leading role in metabolic pathways. Leucine, tryptophan, tyrosine, isoleucine and valine were all significantly up-regulated in glucosinolate biosynthesis.

## Background

Sweetpotato (*Ipomoea batatas* L.), a dicotyledonous plant which belongs to the *Convolvulaceae* family, ranks as the seventh-most important food crop in the world. As a major nutrition organ, storage root (SR) possessed a mass content of starch and photoassimilate. Starch accounts for 50–80% proportion of the dry matter in the SR [1, 2]. Since soluble sugar content is very low in freshly harvested roots during general production process, a certain time of post-harvest storage at 13–15°C is imperative to facilitate starch-sugar interconversion and boost the sweetness to increase the tuberous food quality before sale. It is noticeable that exposure to 5°C for 20 d has been observed to increase sweetness of ‘Kokei 14’ roots, however, this treatment also caused rottenness and high rate of carbohydrate loss [3]. Therefore, a better understanding of biochemical and molecular response mechanisms to chilling stress is essential for extending tuberous crops storage time under low temperature condition.

Compared with model plants, it is more difficult for sweetpotatoes to find out genes implicated in various stress tolerance because of its complicated genetic background. Although some genomic [4, 5] and proteomic [6–8] resources of sweetpotatoes have been available now, these pieces of information are still limited to explicate the molecular mechanism of chilling resistant. With the development of sequencing technique, metabolomics has been considered as a powerful complementary tool to acquire the biological information associated with the metabolites. Metabolites are not only the end-products of expressions of some genes, but also the consequence of interaction between the genome and its milieu. Therefore, it is probable to envisage the functional genomics assembly by connecting gene expression to the metabolomic knowledge [9].

As a chilling-sensitive tropical crop, sweetpotatoes can be irreparably damaged when the temperature drops below 10°C. A main reason for this is oxidative injuries caused by an increased accumulation of reactive oxygen species (ROS) [10–16]. In plants, stress-induced ROS scavenging is usually implemented by both enzymatic and non-enzymatic low molecular metabolic antioxidants [17, 18]. As we all know, the starch content in fresh roots of sweetpotatoes is about 15–30% [8]. Soluble sugar not only serves as substrates for starch production, but also may also function as a signal involved in chilling defense for tubers.

To better explore the proteins and metabolic pathways under chilling condition, we carried out the proteometabolomic profile of fresh sweetpotatoes to clarify the cold stress-responsive mechanism. Integration of proteomic and metabolomic profiles information would give new insights into the molecular functions of tuberous root crops during post-harvest storage. This would provide a basis for future comparative proteomic efforts for this important crop including gene discovery and improvement of chilling stress tolerance.

## Results

### Morphological variations under cold storage

**Fig. 1:**
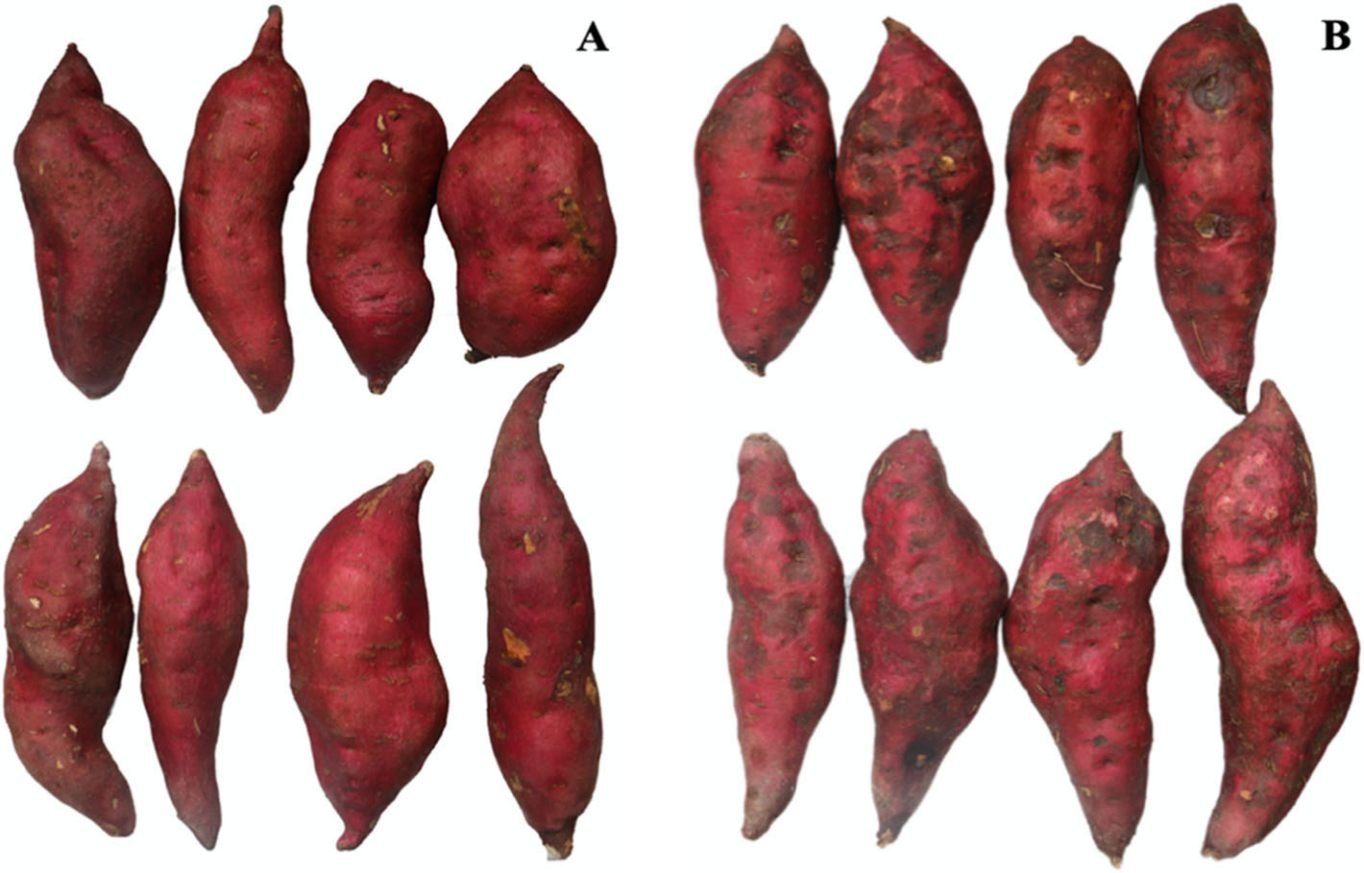
Morphological differences in tuber shape and color during storage at 13 °C (**a**) and 4 °C (**b**) for 14 d

To investigate the effect of chilling stress on the storage of sweetpotatoes, freshy harvested ones (cv. Xinxiang) were stored in the storage chamber of 13 °C (CK) and 4 °C for 14 days (d). As shown in Fig. 1 and Table 1, roots at 13 °C showed no chilling injury (CI) symptoms, while the epidermis of roots exposed to 4 °C (Fig. 1b) were significantly spotted and shriveled than those stored at 13 °C (Fig. 1a). The CI index was also significantly higher than that of control roots. In addition, the water content exhibited significantly decreases under 13 °C, and no differences were found under low temperature (4 °C).

**Table 1:** CI index and water content of sweetpotatoes after storage at different temperatures

Storage time (d)	CI index		Water content (%FW)	
	13 °C	4 °C	13 °C	4 °C
0	0.0 ± 0.0a	0.0 ± 0.0b	64.5 ± 2.5a	64.5 ± 3.1a
14	0.0 ± 0.0a	0.7 ± 0.1a	60.7 ± 1.6b	64 ± 2.7a

### Effects of low-temperature storage on oxidative stress

**Fig. 2:**
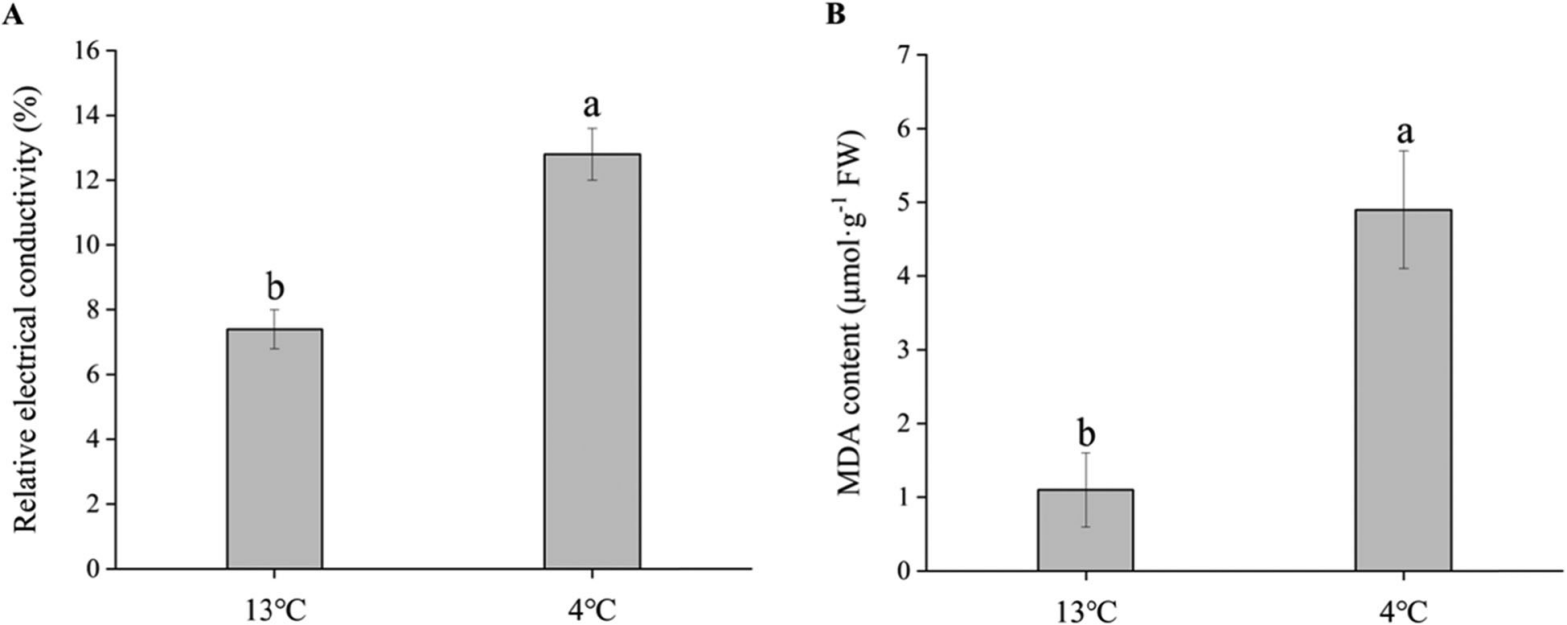
Effects of low-temperature storage on relative electrical conductivity (REC) and MDA content in sweetpotato roots for 14 d. **a** Relative electrical conductivity. **b** MDA content. Vertical bars represent the mean ± SE. Different letters indicate statistically significant differences (*p*<0.05) by LSD test

**Fig. 3:**
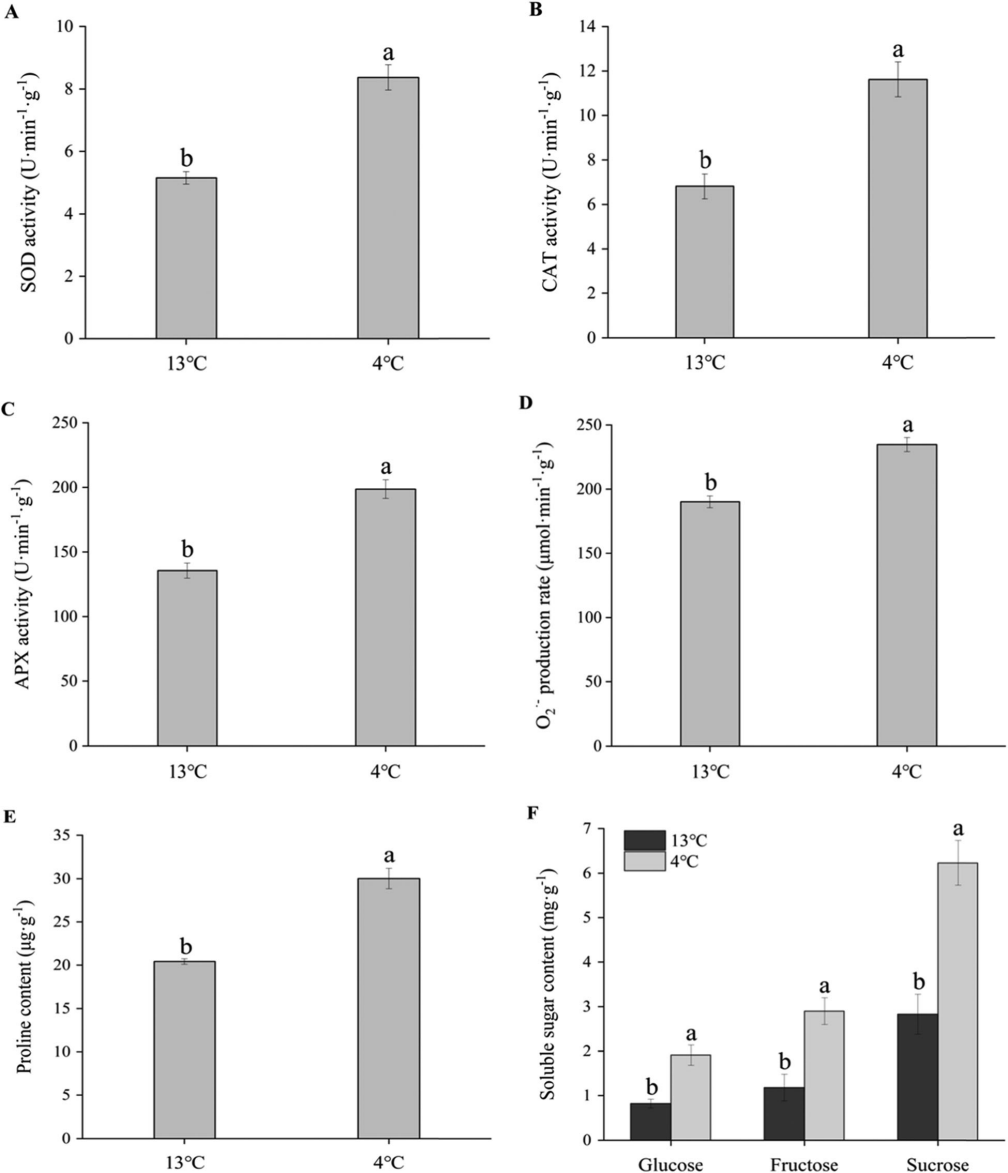
Effect of low-temperature storage on oxidative stress in terms of SOD (**a**), CAT (**b**), APX (**c**) activities, O_2_^−^ producing rate (**d**), proline content (**e**) and soluble sugar content (**f**) such as glucose, fructose, and sucrose in sweetpotatoes for 14 d. Vertical bars represent the mean ± SE. Different letters indicate statistically significant differences (*p*<0.05) by the LSD test

The relative electrical conductivity (REC) level and malondialdehyde (MDA) content were significantly higher in the roots exposed to 4 °C condition than that at 13 °C (Fig. 2). The activities of SOD, CAT, APX, O_2_^.-^ producing rate, proline and soluble sugar contents have been shown in Fig. 3. Similarly, the low temperature (4 °C) significantly increased the activities of antioxidant enzymes (Fig. 3a, b, c) and the production rate of O_2_^.-^ (Fig. 3d). Moreover, chilling stress also enhanced the proline (Fig. 3e), glucose, fructose and sucrose (Fig. 3f) contents. It’s worth mentioning that three types of soluble sugar contents were increased most among above of physiological indexes, by 112.4, 145.6 and 139.4%, respectively.

### Segregation and identification of proteins

Compared to the control, 266 and 158 proteins were found significantly up- and down-regulated by > 1.5 fold, respectively in roots under 4 °C storage (Supplementary Table S2, Additional file 1 and Additional file 2). The protein bands were clear, uniform and not degraded in each lane (Supplementary Figure S1). The molecular masses of identified proteins were distributed 5–275 kDa, with majority of proteins (96%) distributed in the range of < 100 kDa (Supplementary Figure S2). The extracted proteins were suitable for further LC-MS/MS analysis.

### Annotation of DEPs in GO classification, subcellular localization and pathway enrichment

**Fig. 4:**
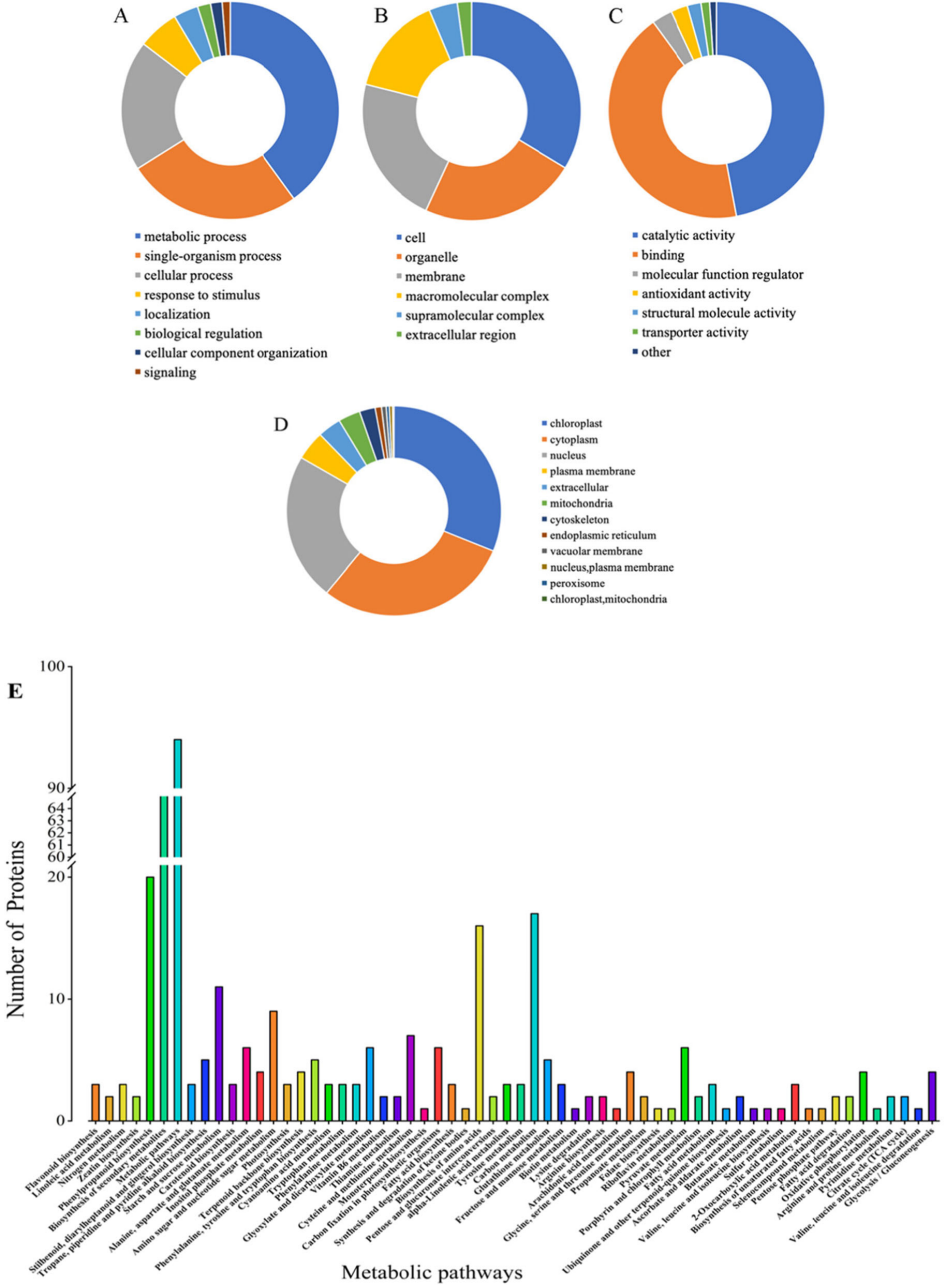
Functional classification, subcellular localization and pathway affiliation of proteins. Identified proteins were categorized according to their gene ontology for their biological processes (**a**), cellular components (**b**), molecular functions (**c**), subcellular localizations (**d**) and association with different metabolic pathways (**e**)

Annotation of differentially expressed protein (DEPs) function and their cellular location is necessary to understand their roles at molecular level (Additional file 3). The results demonstrated that they were grouped into 15 distinct categories. These proteins were mainly implicated in metabolic processes, cellular components, catalytic activities and binding (Fig. 4a, b, c). Most of them were associated with catalytic activities (~ 47%), followed by binding (~ 43%), metabolic process (~ 40%), cell (~ 34%) and organelle (~ 23%).

In addition, the DEPs were delegated based on their presence in a particular compartment (Additional file 4). Most of them were localized in the chloroplast/cytoplasm (~ 30%), followed by nucleus (~ 15%) and plasma membrane (~ 5%) (Fig. 4d).

The identified proteins were further analyzed via KEGG database for interpretation of their involvement in different metabolic pathways (Additional file 5). Most of the DEPs were implicated in pathways related to metabolic pathway (~ 22%), followed by biosynthesis of secondary metabolites (~ 16%), and phenylpropanoid biosynthesis (Fig. 4e).

### DEPs involved in phenylpropanoid biosynthesis

As previously mentioned, most of proteins were involved in metabolic pathway and biosynthesis of secondary metabolites. Phenolic compounds regulated by phenylalanine ammonia lyase (PAL), cinnamyl alcohol dehydrogenase (CAD), Hydroxycinnamoyl transferase (HCT) were listed in Table 2. The *p* value of these proteins was negatively corelated with their significances in phenylpropanoid biosynthesis pathway. Hence, the significance order of DEPs was shikimate>peroxidase4 > 4-coumarate-CoA ligase>Cytochrome P450 (cytochrome P450 monooxygenases) > PAL>CAD.

**Table 2:** Part of DEPs participated in phenylpropanoid biosynthesis

Differentially expressed proteins	*p* value
Phenylalanine ammonia lyase	5.6 × 10^−9^
Cinnamyl alcohol dehydrogenase	4.3 × 10^−8^
Peroxidase 4	1 × 10^−32^
Cytochrome P450	3.7 × 10^−13^
4-coumarate-CoA ligase	1.1 × 10^−16^
shikimate O-hydroxycinnamoyl transferase	1 × 10^−32^

### Differential multiple of the DEPs participated in starch and sucrose metabolism

**Fig. 5:**
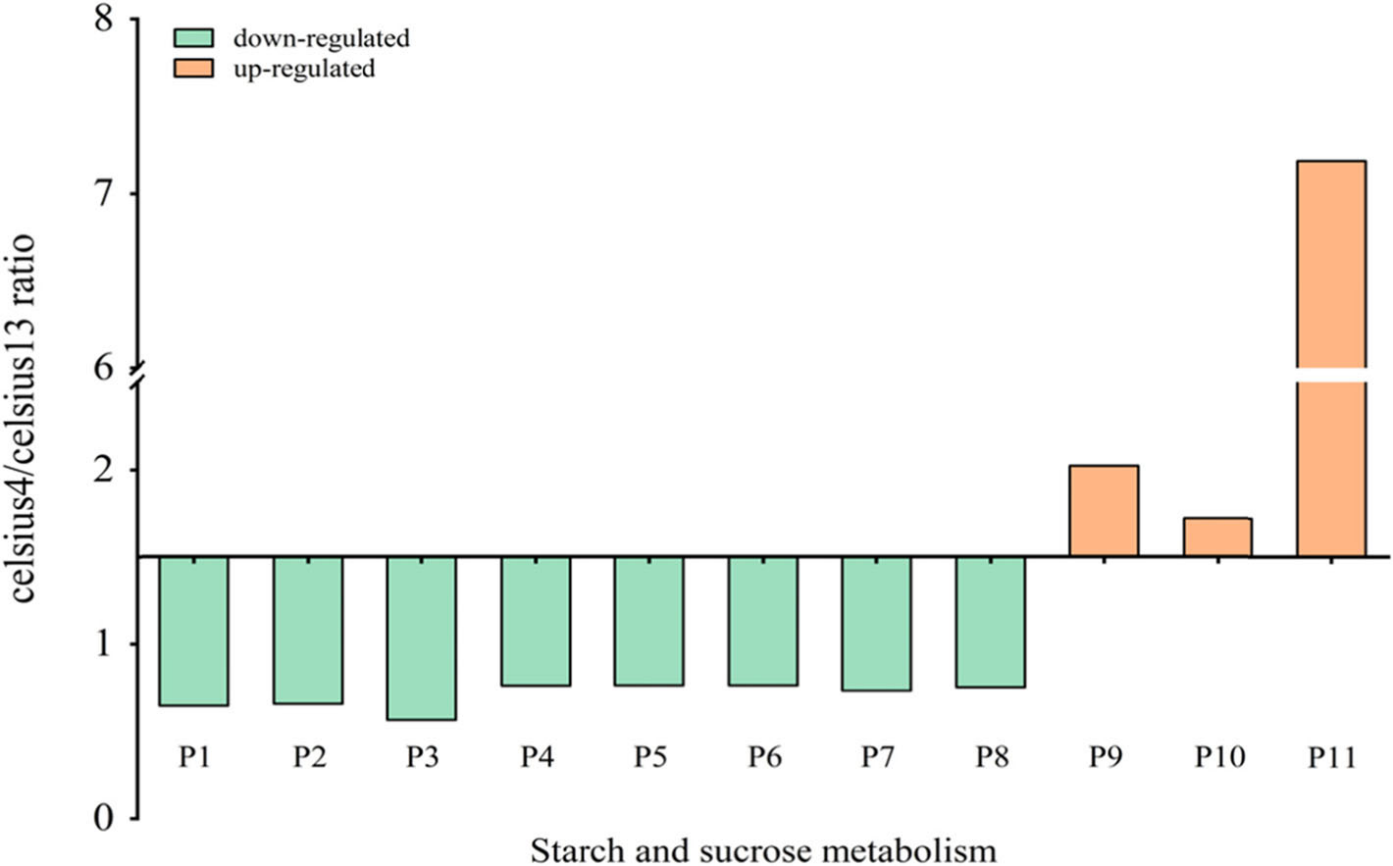
Differential multiple of the differentially expressed proteins (DEP) participated in starch and metabolism. P1: Glucose-1-phosphate adenylyltransferase (large subunit); P2: β-xylosidase/α-arabinofuranosidase 2; P3: β-glucosidase 12; P4: Glucose-1-phosphate adenylyltransferase (small subunit); P5: Sucrose synthase 6; P6: Glucan endo-1,3-β-glucosidase 6; P7: 4-α-glucanotransferase; P8: Isoamylase 3; P9: α-amylase; P10: Probable fructokinase 7; P11: Sucrose synthase

As compared to the roots stored at 13 °C, there were 11 DEPs participated in starch and sucrose metabolism under 4 °C (Fig. 5). The filtered *p* value matrix (*p* < 0.05) transformed by the function x = −lg (*p* value) was conduct to evaluate the celesius4/celesius13 ratio, which was positively corelated with the differential multiple of DEPs. Three proteins (x > 1.5) were up regulated, while others (x < 1.5) presented an opposite trend in this metabolic pathway. The ratio of sucrose synthase (P11) and β-glucosidase (P3) was 7.19 and 0.56, significantly higher and lower than other proteins, respectively (Fig. 5).

### Functional network of the DEPs in starch and sucrose metabolism

**Fig. 6:**
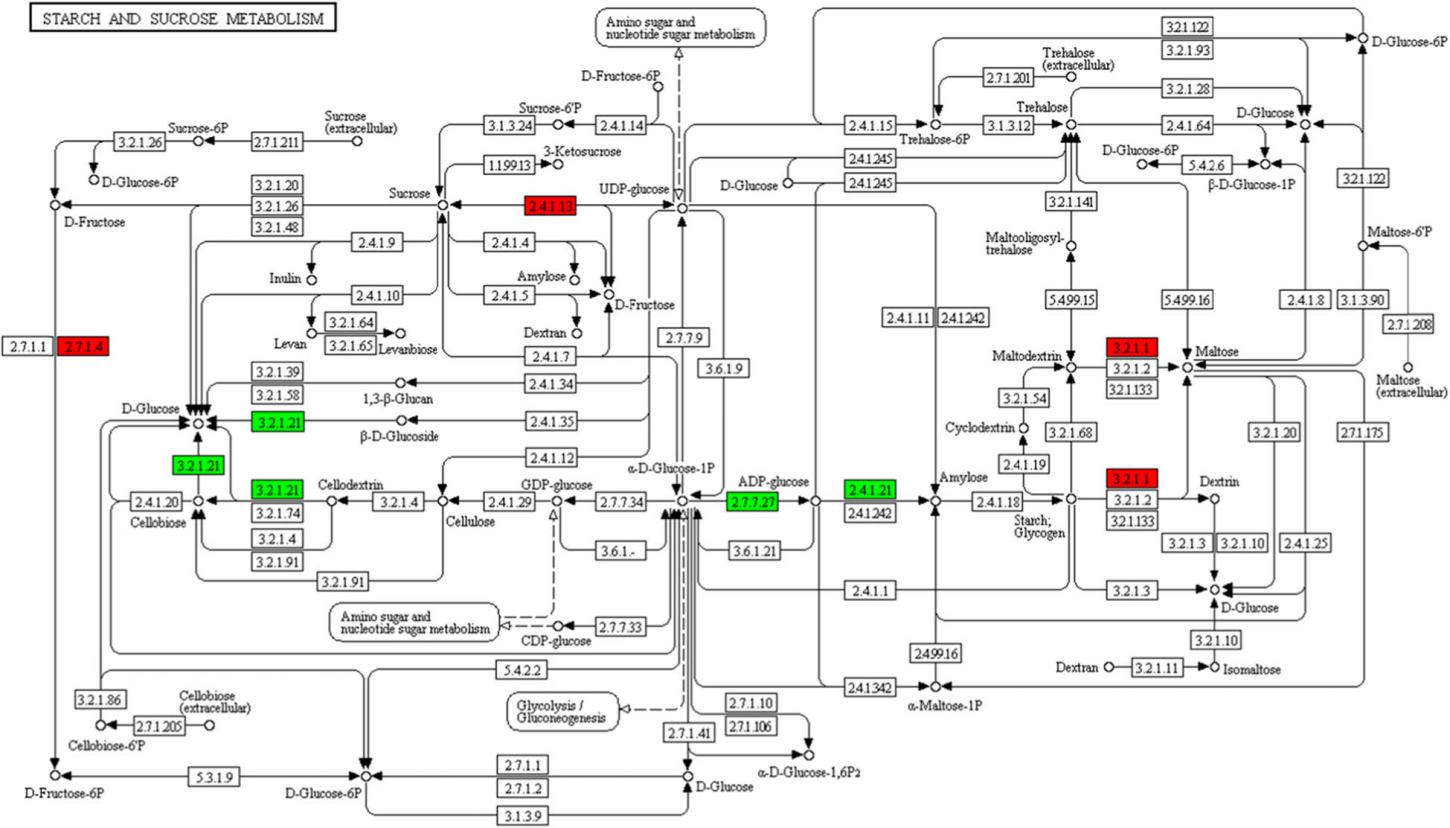
Changes of differentially expressed proteins (DEPs) involved in starch and sucrose metabolism of sweetpotato roots under cold stress. The significantly up-(red) and down-regulated (green) expressed proteins are demonstrated. EC: 3.2.1.1, EC: 2.4.1.13 and EC: 2.7.1.4 proteins (red) were α-amylase, sucrose synthase and fructokinase, respectively. EC: 3.2.1.21, EC: 2.7.7.27 and EC: 2.4.1.21 proteins (green) were β-glucosidase, glucose-1-phosphate adenylyl-transferase and starch synthase, respectively

The functional network under chilling stress for roots was illustrated in Fig. 6. There were three up- and three down-regulated DEPs. α-amylase (EC: 3.2.1.1, red), associated with starch metabolism and carbohydrate digestion or absorption, was significantly up-regulated when maltodextrin or starch was hydrolyzed to maltose. Furthermore, it was homologous with K01177 (β-amylase: EC: 3.2.1.2), K05992 (maltogenic α-amylase: EC:3.2.1.133) in terms of the orthology analysis. Similarly, both of EC: 2.4.1.13 (sucrose synthase) and EC: 2.7.1.4 (fructokinase) were significantly up-regulated in amino and nucleotide sugar metabolism. On the other hand, EC: 3.2.1.21, EC: 2.7.7.27 and EC: 2.4.1.21 proteins, named as β-glucosidase, glucose-1-phosphate adenylyl-transferase and starch synthase, respectively, were significantly down-regulated in starch and sucrose metabolism pathway. They were mainly involved in phenylpropanoid biosynthesis, biosynthesis of starch and secondary metabolites as well as polysaccharide accumulation. The degradation of starch into soluble sugar can not only boost the sweetness, but also significantly improve the resistance to chilling stress.

### Metabolome profiling and its fold change analysis

**Fig. 7:**
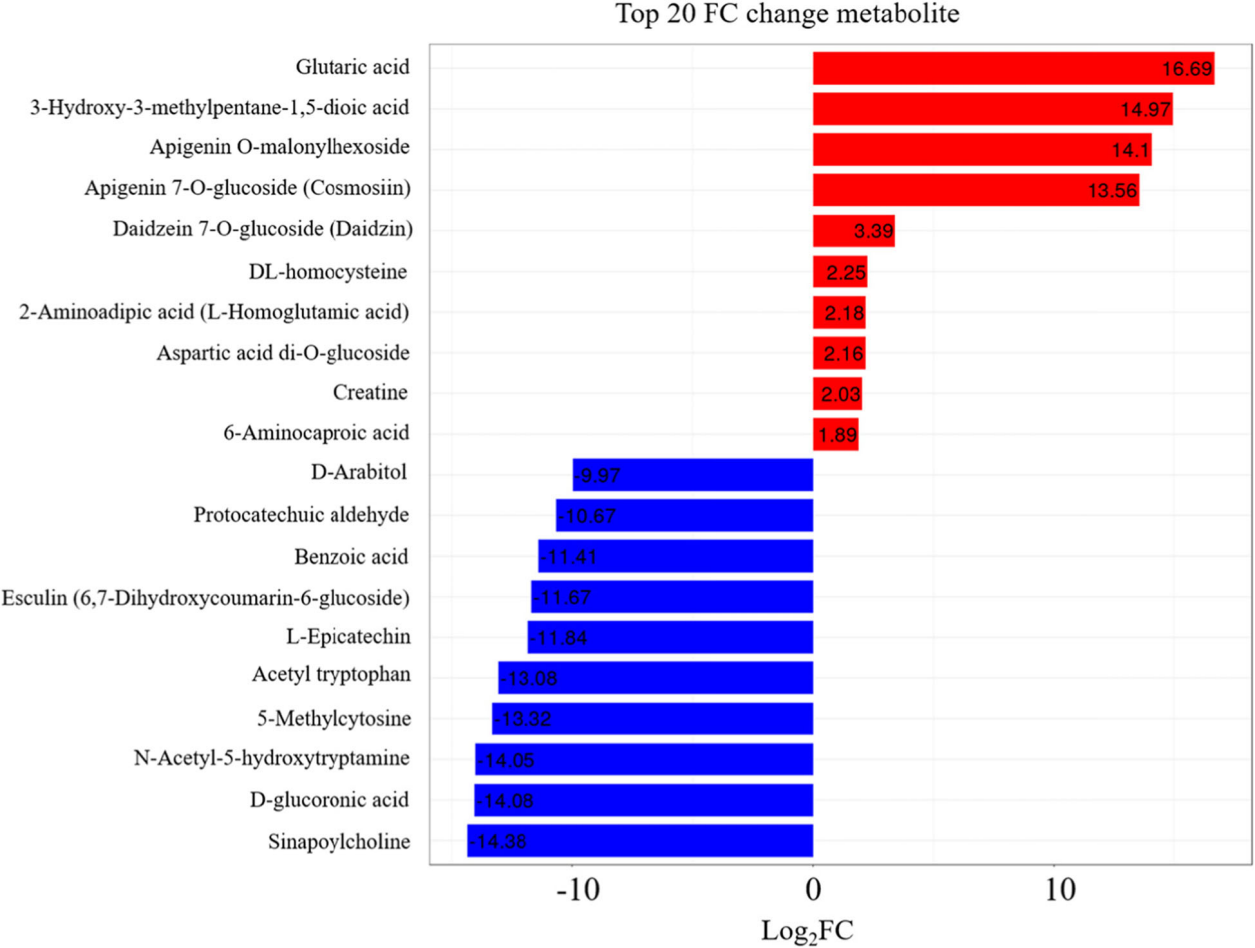
Significant fold changes of the metabolites in sweetpotato roots under chilling stress as compared to them under control. Red and blue lines represent up- and down-regulated metabolites, respectively

The metabolome profiling of sweetpotato tubers led to the identification of 76 differentially expressed metabolites (DEMs) in the roots stored at 4 °C as compared to them at 13 °C. There were 31 up- and 45 down-regulated metabolites (Supplementary Table S3 and Additional file 6). The absolute value level of fold change (FC) was closely related to significance of the metabolic component. The results (Fig. 7) showed that the absolute Log_2_FC values of 4 components in up-regulated metabolites were more than 10.00, including glutaric acid (16.69), followed by 3-hydroxy-3-methylpentane-1,5-dioic acid (14.97), apigenin O-malonylhexoside (14.1) and apigenin 7-O-glucoside (cosmosiin) (13.56). Nevertheless, the absolute values of 9 components were more than 10 in down-regulated DEMs, namely sinapoylcholine (14.38), D-glucoronic acid (14.08), N-acetyl-5-hydroxytryptamine (14.5), 5-Methylcytosine (13.32) etc. The metabolic activities of a large proportion of identified components dropped off in roots under 4 °C.

### Screening and distribution of DEMs in roots under chilling stress

**Fig. 8:**
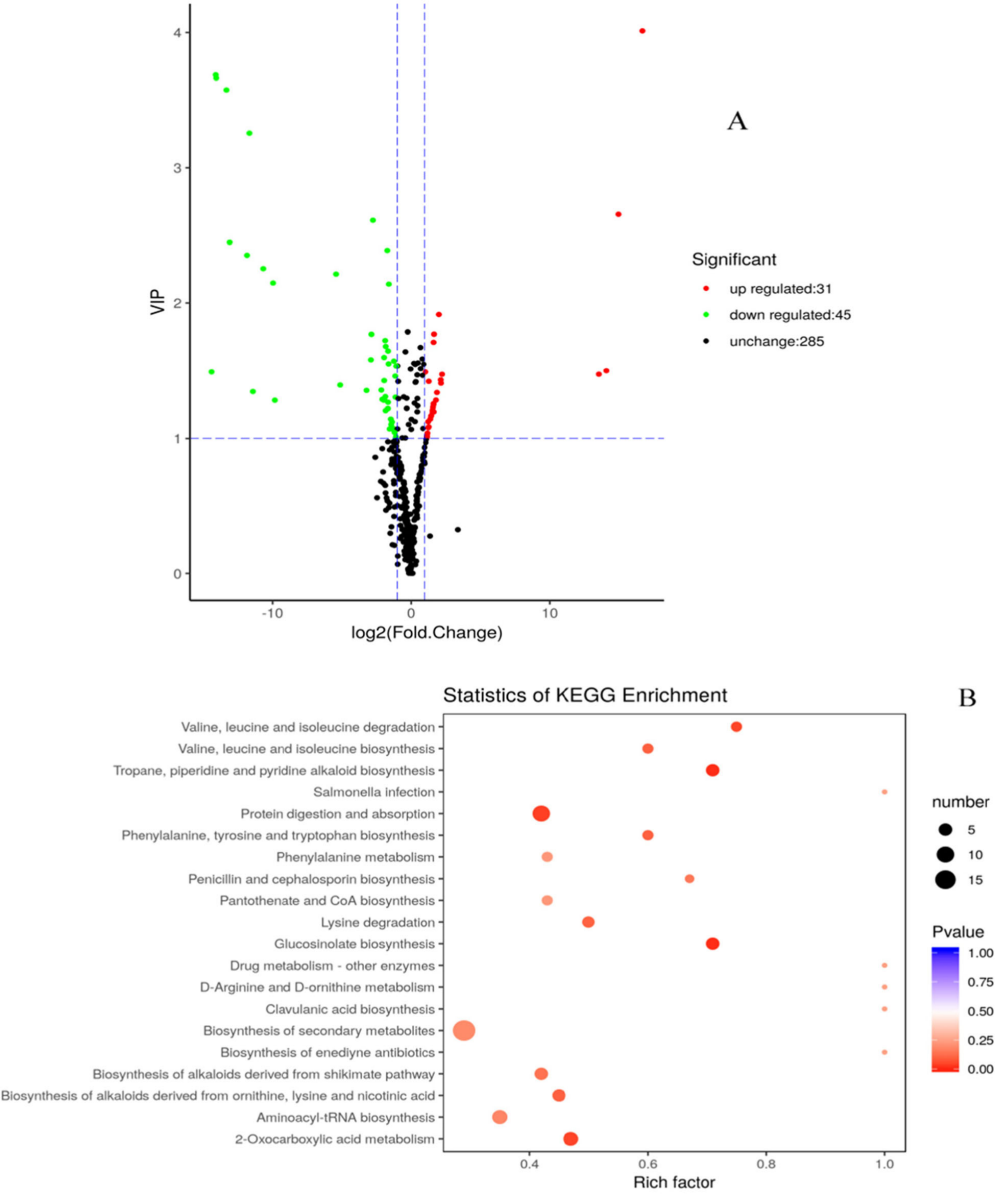
The volcano plots and statistics of KEGG pathway enrichment of significantly differential expressed metabolites (DEMs) were demonstrated. In the volcano plots, red, green and black dots represent up-, down-regulated and insignificant changed metabolites, respectively (**a**). The dimension of dots indicates the amount of the DEMs. The color (*P*-value) explained the significance of DEM. Rich factor means the ratio of the number of the DEMs to the total number of them detected in the corresponding pathway (**b**)

Compared to the absolute value level of fold change, Variable Importance in Project (VIP) value (> 1) was extremely associated with the significance of metabolic compound in the corresponding class. All the identified DEMs were categorized into 20 classes. Most of them (~ 33%) were belonging to nucleotide, its derivates and amino acid derivatives group. On the basis of VIP and Log_2_FC value, the results (Table 3) illustrated that most of components were down-regulated except 3-hydroxy-3-methylpentane-1,5-dioic acid and glutaric acid. The VIP and Log_2_FC value of glutaric acid, belonged to organic acids, were the highest (4.01 and 16.69, respectively), followed by D-glucoronic acid (3.69 and 14.08), N-acetyl-5-hydroxytryptamine (3.66 and 14.05) and 5-Methylcytosine (3.58 and 13.32) (Table 3 and Fig. 8a). Carbohydrates were represented by D-glucoronic acid, which was an important member of sugar metabolism.

**Table 3:** Screening of differential expressed metabolic components

Compounds	Class	VIP	Log_2_FC	Type
Glutaric acid	Organic acids	4.01	16.69	up
D-glucuronic acid	Carbohydrates	3.69	14.08	down
N-acetyl-5-hydroxytryptamine	Tryptamine derivatives	3.66	14.05	down
5-Methylcytosine	Nucleotide and its derivates	3.58	13.32	down
Esculin	Coumarins	3.26	11.67	down
3-Hydroxy-3-methylpentane-1,5-dioic acid	Amino acid derivatives	2.66	14.97	up
O-sinapoyl quinic acid	Quinate and its derivatives	2.61	2.75	down
Acetyl tryptophan	Amino acid derivatives	2.45	13.08	down
Sinapic acid	Hydroxycinnamoyl derivatives	2.39	1.72	down
L-Epicatechin	Catechin derivatives	2.35	11.84	down
Protocatechuic aldehyde	Catechin derivatives	2.26	10.67	down
Pantetheine	Vitamins	2.21	5.40	down
D-arabitol	Alcohols and polyols	2.15	9.97	down

Furthermore, KEGG pathway enrichment was conducted in terms of their *P*-values and rich factors. P-value and rich factor had negative and positive correlation with enrichment significance of metabolic compounds, respectively. The P-value of glucosinolate biosynthesis, tropane, piperidine and pyridine alkaloid biosynthesis (9.94 × 10^− 3^) was obviously lower than protein digestion and absorption (3.56 × 10^− 2^) (Table 4 and Fig. 8b).

**Table 4:** KEGG pathway enrichment of significantly DEMs

KEGG pathway enrichment	*P*-value	Compounds
glucosinolate biosynthesis	9.94 × 10^−3^	Leu; Try; Tyr; Ile; Val
tropane, piperidine and pyridine alkaloid biosynthesis	9.94 × 10^−3^	Putrescine; piperidine; pipecolic acid; Ile; Lys
protein digestion and absorption	3.56 × 10^−2^	Putrescine; piperidine; Indole; Val; Ile; Tyr; Try; Arg; Lys; Leu

*Abbreviation*: *Leu* Leucine, *Try* Tryptophan, *Tyr* Tyrosine, *Ile* Isoleucine, *Val* Valine, *Arg* Arginine, *Lys* Lysine

### Network of the differential metabolic compounds in glucosinolate biosynthesis

**Fig. 9:**
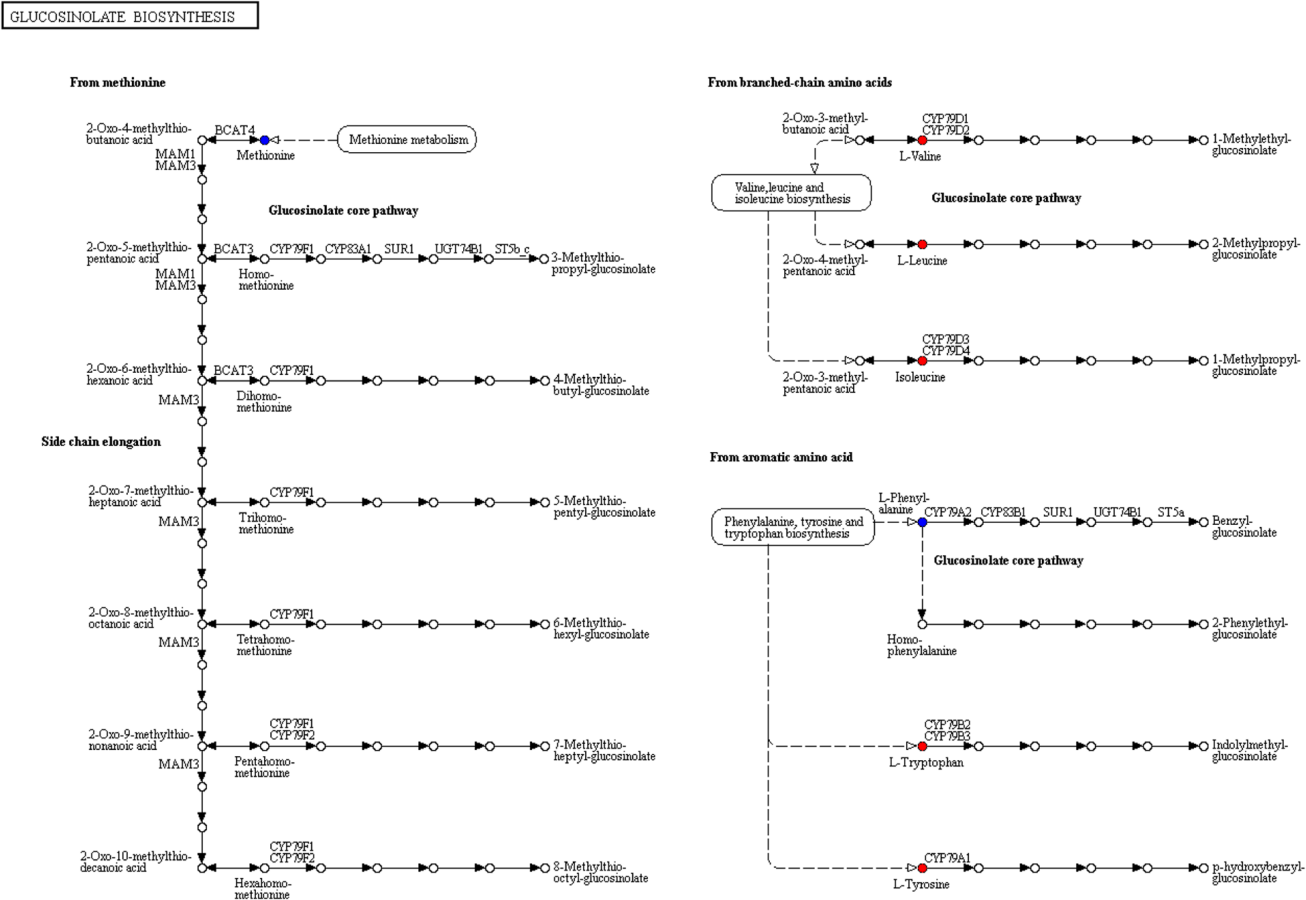
Differential expressed metabolic components in glucosinolate biosynthesis. Red and blue dots represent up-regulated and insignificant changed compounds, respectively

As previously mentioned, glucosinolate biosynthesis, comprised of amino acid such as leucine (Leu), tryptophan (Try), tyrosine (Tyr), isoleucine (Ile) and valine (Val), was significant in metabolic pathways for increasing the chilling tolerance of sweetpotato roots. The glucosinolate can be synthesized from methionine, branched-chain amino acids or aromatic amino acids process (Fig. 9). Leu, Ile and Val were involved in branched-chain amino acids. Try and Tyr were imperative for aromatic amino acids pathway. All these amino acids were significantly up-regulated in glucosinolate biosynthesis (Fig. 9).

## Discussion

### ROS scavenging and osmotic adjustment substances

The growth and productivity of higher plants are severely limited by environmental stresses including low-temperature, drought and salinity. MDA content and ion leakage are indicators of membrane damage caused by chilling stress. Gill et al. [19] described that excess ROS resulted in rise of MDA, membrane leakage and DNA breakdown which cause severe damage to plant cell. Plants have evolved in the presence of ROS and have acquired dedicated pathways to protect themselves against oxidative damage and fine modulation of low levels of ROS for signal transduction [20–24]. The enzymatic systems of ROS scavenging mechanisms mainly include SOD, POD, CAT and APX [25]. The expressions of intracellular genes *CuZnSOD* and *swAPX1* were significantly correlated with low temperature stress (4 °C) [26]. SOD is able to rapidly convert ·OH to H_2_O_2_, and the generated H_2_O_2_ is then converted to water and dioxygen by CAT and APX [27–29]. However, abiotic stress resistance has been increased in rice mutants with double silenced for cytosolic APXs gene (APX1/2 s) [30]. In our research, L-ascorbate peroxidase 3 and catalase were down-regulated during 4 °C storage (Additional file 2), nevertheless the CAT/APX activities (Fig. 3b, c) were increased. This discrepancy between proteomic and biochemical data may be explained by higher H_2_O_2_ levels and increased ascorbate redox states, which enhanced the CAT/APX activity indirectly.

Induction of osmoprotectants biosynthesis is another type of the plant response to low temperature. Several studies suggested that increased abiotic stress tolerance was obtained by introducing simple metabolic traits from other organisms such as the production of trehalose and proline [31]. Recent data suggested that overexpression of *DlICE1* (*Dimocarpus longan* L.) in transgenic *Arabidopsis* conferred enhanced cold tolerance via increased proline content and antioxidant enzyme such as SOD, CAT, APX [32]. In our research, the low temperature (4 °C) significantly increased the activities of antioxidant enzymes (Fig. 3a, b, c), the producing rate of O_2_^.-^ (Fig. 3d) and proline content (Fig. 3e) as compared to the control (13 °C). Thus, less damage from membrane lipid peroxidation enabled the sweetpotato roots to continue normal metabolism under low-temperature condition, contributing to their higher cold tolerance.

### The role of phenolics compounds and glucosinolate biosynthesis under abiotic stress

Phenylpropanoids are a group of secondary metabolites synthesized from the amino acid phenylalanine [33, 34]. In plants, the phenylpropanoid pathway underlying abiotic stress tolerance is tightly connected with physiological and molecular mechanisms. Phenolic accumulation is usually activated when plants face multiple abiotic stresses [35]. Increased phenolic levels play crucial role in plants protection against chilling stress [36]. Gao et al. [37] confirmed that stimulated phenolic biosynthesis was owing to the enhanced expression of PAL, CAD and HCT by carrying out the experiments with peach under low-temperature stress. In our research, the phenylpropanoid biosynthesis, a main metabolic pathway, were up-regulated by lots of proteins, especially HCT, PAL and CAD (Fig. 4e and Table 2). Thus, our results were consistent with the previeous research. More importantly, the roots decay was not found (Fig. 1 and Table 1) may be due to enhanced thickness of plant cell walls generated by phenolic accumulation, which is beneficial for the prevention of chilling injury [38, 39].

Glucosinolates mainly function as defense molecules [40]. They are also known as mustard oil glucosides. Until now, more than 100 glucosinolates have been found in plants. In terms of their precursor amino acids, glucosinolates can be categorized into indole glucosinolates, aliphatic glucosinolates and aromatic glucosinolates, which were derived from Trp, from Ala, Leu, Ile, Val/Met, and from Phe and Tyr, respectively [41]. These results were consistent with our research (Table 4 and Fig. 9). Previous studies demonstrated that the accumulation of glucosinolate biosynthetic intermediates can limit the production of phenylpropanoids in two Arabidopsis mutants of *ref2* and *ref5* [42, 43]. However, it seems that there was no obviously crosstalk between phenylpropanoids and glucosinolates biosynthesis.

### Sugar as antioxidants in sweetpotato roots

Sweetpotato has been known as one of the highest starch producing crops due to their higher sink strength [44]. Soluble sugars were linked with the production rates of ROS by regulating its producing metabolic pathways, such as mitochondrial respiration or photosynthesis [45]. Starch content has significantly negative relationship with sucrose in tuber crops, because the catabolism of starch been impaired with respiratory rate decrease in low storage temperature. In our study, three types of soluble sugar contents were obviously increased (Fig. 3f). Moreover, α-amylase, β-amylase, sucrose synthase and fructokinase were significantly up-regulated, which boosted the sweetness of the tuber roots under cold temperature (Figs. 5 and 6). These results confirmed that sweetness could enhance the chilling stress tolerance of sweetpotato roots.

Nevertheless, the concept ‘sugar as antioxidant’ has been put forward in recent years [46], and some genes encoding different sugar compounds were confirmed to enhance low temperature tolerance in petunia, tobacco and rice [47–51]. It is more and more recognized that water-soluble carbohydrates (glucose, fructose and sucrose) are regarded as key regulators in plant responses to abiotic/biotic stress. High antioxidant protection was maintained by an accumulation of increased soluble sugars under drought stress in *Arabidopsis thaliana* leaves [52]. In addition, low temperature can affect the metabolic activities and cause osmotic stress in plants. Cell turgor is able to be stabilized by soluble carbohydrates [53]. Hence, both simple sugars and polysaccharides are necessary for plant survival under stress conditions [54, 55].

## Conclusions

In summary, our proteomic and metabolic profile analysis of sweetpotatoes reveal that the CI index, REC and MDA content in roots stored at 4 °C were much higher than them at 13 °C. Low-temperature storage condition significantly enhanced the activities of SOD, CAT, APX, O_2_^.-^ producing rate, proline and especially soluble sugar contents. Most of DEPs were implicated in pathways related to metabolic pathway, especially phenylpropanoids and followed by starch and sucrose metabolism. α-amylase, sucrose synthase and fructokinase were significantly up-regulated in starch and sucrose metabolism, while β-glucosidase, glucose-1-phosphate adenylyl-transferase and starch synthase were opposite. Interestingly, there was discrepancy between proteomic (L-ascorbate peroxidase 3 and catalase) and biochemical (CAT/APX enzyme activity) data, which may be owing to higher H_2_O_2_ levels and increased glutathione and ascorbate redox states, which enhanced the CAT/APX activity indirectly. Moreover, glucosinolate biosynthesis played a leading role in metabolic pathways. Leucine, tryptophan, tyrosine, isoleucine and valine were all significantly up-regulated in glucosinolate biosynthesis. These results would expand our knowledge of the proteome and metabolome about the chilling tolerance of sweetpotatoes.

## Methods

### Plant materials and storage condition

Sweetpotatoes (*I. batatas* L. cv. Xinxiang), obtained from Zhejiang Academy of Agricultural Sciences of China (supplementary Table S1), were grown in the greenhouse at 25–30 °C under a long-day photoperiod (16/8 h, light/dark) according to standard agricultural practices in 2018. The mature roots (average weight 100-120 g) were selected with the same size. They had no physical injury or bacterial infection. Then, they were divided randomly into two groups with three replicates and stored in Temperature Humidity Chamber of 4 °C and 13 °C (CK) for 14 days, respectively (Laifu MJX-280H, China). After storage, the tubers were sliced to 1 mm thickness, put into liquid nitrogen and stored at − 80 °C for further analysis [26].

### Estimation of chilling injury index

The apparent condition of surface pitting, dark watery patches, and internal tissue browning were the typical CI symptoms of tuberous roots [26, 56]. Ten roots for each replicate were chosen for CI evaluated visually. The CI index calculation was according to Li et al. [26].

### Relative electrical conductivity and malondialdehyde content assays

The relative electrical conductivity (REC) was measured as previously described with some modification [57]. The REC calculation was according to Hu et al. [58]. The MDA content was determined by the thio-barbituric acid method [58].

### Determination of antioxidant enzyme, the rate of O_2_^·-^ production and proline content

For enzyme activities, the fresh roots (0.1–0.5 g) were homogenized in 10 ml of 50 mM precooled potassium phosphate buffer (PBS; pH 7.8) under ice cold conditions [59]. Superoxide anions (O_2_^·-^) producing rate was determined according to Jiang & Zhang [60] with some modifications. Superoxide dismutase (SOD), catalase (CAT) and ascorbate peroxidase (APX) activity was determined according to Dhindsa & Matowe [61], Aebi [62], and Nakano & Asada [63], respectively. Proline content was measured according to Bates et al. [64].

### Determination of soluble sugar composition

High performance liquid chromatography (HPLC) was used to determine the composition of soluble sugars (glucose, fructose and sucrose) in roots. The procedure of HPLC was in terms of Li et al. [26].

### Protein extraction and 1-DE SDS-PAGE

The flesh of sweetpotato was grinded by liquid nitrogen into fine powder and four volumes of lysis buffer (8 M urea, 1% Triton-100, 10 mM dithiothreitol and 1% Protease Inhibitor Cocktail) were added to sonication extract three times on ice using a high intensity ultrasonic processor. The ultrasonic time was 3s of ultrasound and 6s of pause, with a total of 20 cycles. The electrophoresis of protein samples was performed with 12% SDS PAGE gels and stained by Coomassie Blue R-250.

### LC-MS/MS of digested peptides

The tryptic peptides were loaded onto a home-made reversed-phase analytical column (15 cm length, 75 μm i.d.). The digested peptide was subjected to NSI source followed by tandem mass spectrometry (LC-MS/MS) in Q Exactive™ Plus (Thermo Scientific). Peptides were then selected for MS/MS using NCE set as 28 and the fragments were detected at a resolution of 17,500 in the Orbitrap [65].

### Database searching of proteins

The MS/MS data generated were processed using Maxquant search engine (v.1.5.2.8) in uniport Toxoplasma gondii database. Tandem mass spectra were searched against Vert_tom_20141002 database (117,248 entries). Carbamidomethyl on Cysteine was specified as fixed modification and oxidation on Met was specified as variable modifications [66].

### Bioinformatics analysis

The Gene Ontology (GO) annotation proteome was derived from the UniProt-GOA database (http://www.ebi.ac.uk/GOA/). Identified proteins domain functional description were annotated by InterProScan (http://www.ebi.ac.uk/interpro/) based on protein sequence alignment method used InterPro domain database. The KEGG (Kyoto Encyclopedia of Genes and Genomes) database was used to annotate protein pathway. Localization of proteins was predicted with wolfpsort software (PSORT/PSORT II). For functional enrichment, a two-tailed Fisher’s exact test was conducted to test the enrichment of the DEPs against all identified proteins.

### Metabolome profiling of tubers

The tubers sliced to 1 mm thickness was crushed using a mixer mill (MM400, Retsch) with a zirconia bead for 1.5 min at 30 Hz. 100 mg flesh powder was extracted overnight at 4 °C with 1.0 ml 70% aqueous methanol. The sample extracts were analyzed using an LC-ESI-MS/MS system (HPLC, Shim-pack UFLC SHIMADZU CBM30A, MS, Applied Biosystems 6500 Q TRAP). The metabolomics approach was according to Yan et al. [67] with some modification.

### Protein and metabolite data analysis

Each metabolite was searched on metware database (MWDB) (https://www.metware.cn). PubChem (https://pubchem.ncbi.nlm.nih.gov/) was used for their classifications. Significance analysis of possible comparisons (*p* value) were tested at *p* < 0.05. The Filtered *p* value matrix transformed by the function x = −lg (*p* value) was used to evaluate the ratio of celesius4 to celesius13, which was positively correlated with the expression multiple of the DEP (x > 1.5). The analysis of variance and mean separation of all metabolites were performed with Analyst 1.6.1, Partial Least Squares-Discriminant Analysis (PLS-DA) and Orthogonal Partial Least Squares-Discriminant Analysis (OPLS-DA) model. Figures were drawn by origin2018.

## Supplementary information

**Additional file 1.** The peptides information of proteins identified in *I*. *batata* roots stored at 4 °C compared to tubers under 13 °C (CK) were subjected to NSI source followed by tandem mass spectrometry (MS/MS) in Q ExactiveTM Plus (Thermo) coupled online to the UPLC.

**Additional file 2.** Protein description of DEPs in *I*. *batata* roots by > 1.5 fold.

**Additional file 3.** Functional classification of Gene ontology (GO) annotation of *I*. *batata* proteins. Proteins were assigned into three categories: biological process, cellular components and molecular functions.

**Additional file 4.** Subcellular location of differentially expressed proteins (DEPs) identified by > 1.5 fold in *I*. *batata* roots under cold stress (4 °C) compared to CK.

**Additional file 5.** KEGG annotations of DEPs in *I*. *batata* roots under cold stress (4 °C) compared to CK.

**Additional file 6.** Differentially expressed metabolites (DEMs) information in *I*. *batata* roots under cold stress (4 °C) compared to CK.

**Additional file 7: Table S1.** Information of sweetpotato materials. **Table S2.** Information of differentially expressed proteins. **Table S3.** Number of differentially expressed metabolisms. **Figure S1.** SDS-PAGE of total proteins extracted from root tuber of *Ipomoea batatas* L.. (30 μg total proteins each lane). **Figure S2.** Distribution of proteins according to molecular weights.

## Data Availability

The datasets generated during the current study are available in the PRIDE partner repository with the accession number PXD017728, https://www.ebi.ac.uk/pride/login, and they are available from the corresponding author upon reasonable request (Huqing Yang, yanghuqing@sohu.com).

## Abbreviations

APX: Ascorbate peroxidase
Arg: Arginine
CAD: Cinnamyl alcohol dehydrogenase
CAT: Catalase
CI: Chilling injury
DEMs: Differentially expressed metabolites
DEPs: Differentially expressed proteins
FC: Fold change
GO: Gene Ontology
HCT: Hydroxycinnamoyl transferase
Ile: Isoleucine
KEGG: Kyoto Encyclopedia of Genes and Genomes
Leu: Leucine
Lys: Lysine
MWDB: Metware database
OPLS-DA: Orthogonal Partial Least Squares-Discriminant Analysis
PAL: Phenylalanine ammonia lyase
PLS-DA: Partial Least Squares-Discriminant Analysis
ROS: Reactive oxygen species
O_2_: Superoxide anions
SOD: Superoxide dismutase
Try: Tryptophan
Tyr: Tyrosine
Val: Valine
VIP: Variable Importance in Project
