# Supplementary material for: Proteomic and metabolic profile analysis of low-temperature storage responses in Ipomoea batata Lam. tuberous roots
Source: BMC Plant Biol. 2020 Sep 21;20:435. doi: 10.1186/s12870-020-02642-7 (PMC7507648; doi:10.1186/s12870-020-02642-7)
Supplement: Supplementary file 7 — Additional file 7: Table S1. Information of sweetpotato materials. Table S2. Information of differentially expressed proteins. Table S3. Number of differentially expressed metabolisms. Figure S1. SDS-PAGE of total proteins extracted from root tuber of Ipomoea batatas L.. (30 μg total proteins each lane). Figure S2. Distribution of proteins according to molecular weights. [file 12870_2020_2642_MOESM7_ESM.docx]

**Supplementary data**

Table S1 Information of sweetpotato materials

| Taxon | Variety | Herbarium | Voucher | Geographic origin | identifier |
| --- | --- | --- | --- | --- | --- |
| *Ipomoea batata* L. | Xinxiang | Zhejiang Academy of Agricultural Sciences | 2007001 | Hangzhou, Zhejiang, China | Liehong Wu |

Table S2 Information of differentially expressed proteins

|  | Up-regulation (>1.5) | Down-regulation (<1.5) |
| --- | --- | --- |
| 4℃-VS-13℃ | 266 | 158 |

Table S3 Number of differentially expressed metabolisms

|  | All sig diff | Down-regulation | Up-regulated |
| --- | --- | --- | --- |
| 4℃-VS-13℃ | 76 | 45 | 31 |


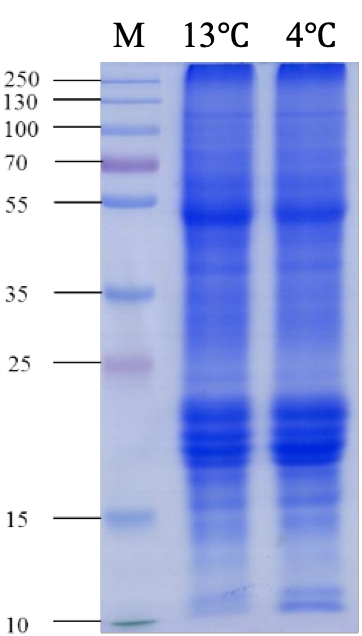


Fig. S1. SDS-PAGE of total proteins extracted from root tuber of *Ipomoea batatas* L.. (30 μg total proteins each lane)


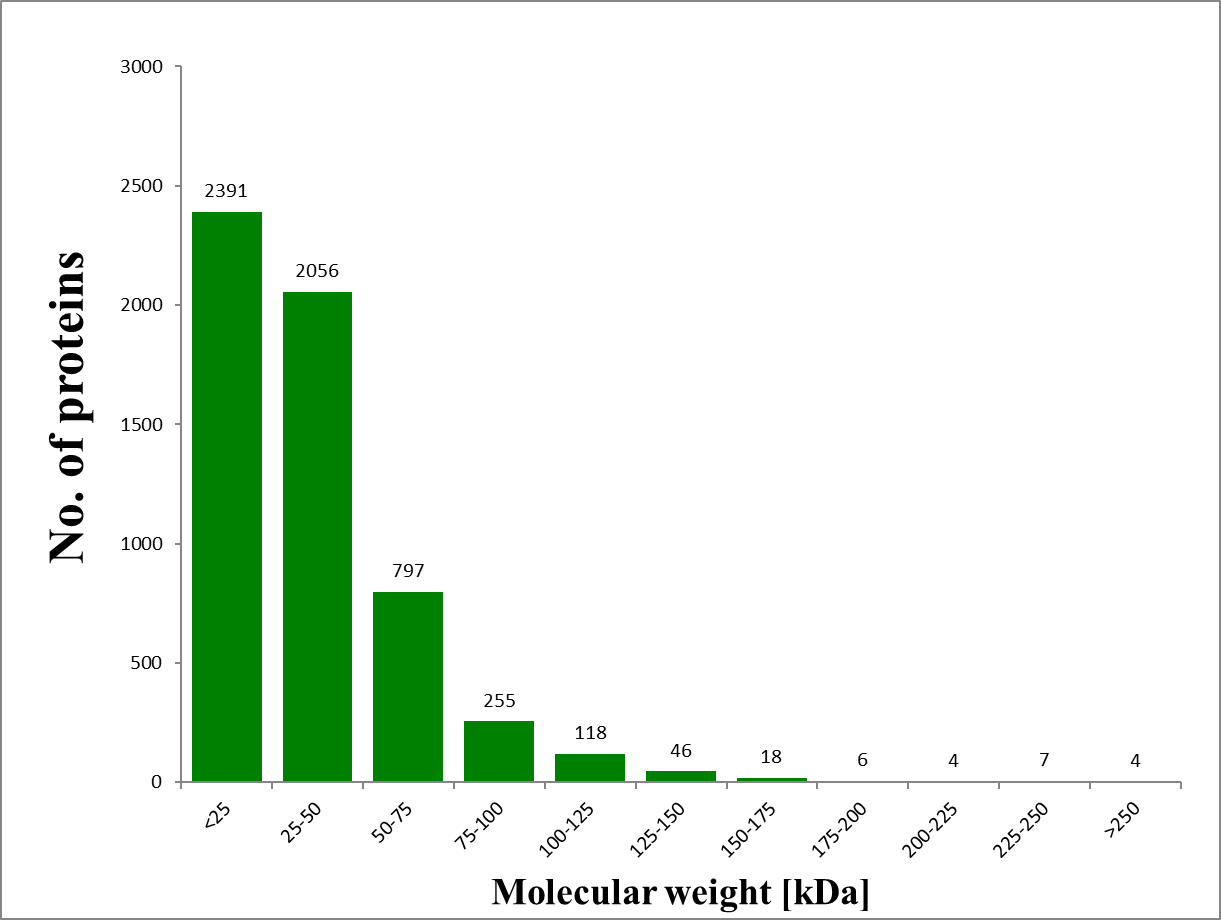


Fig. S2. Distribution of proteins according to molecular weights
